# Associations of visceral fat area and physical activity levels with the risk of metabolic syndrome in postmenopausal women

**DOI:** 10.1007/s10522-017-9693-9

**Published:** 2017-03-18

**Authors:** Izabela Zając-Gawlak, Barbara Kłapcińska, Aleksandra Kroemeke, Dariusz Pośpiech, Jana Pelclová, Miroslava Přidalová

**Affiliations:** 1 The Jerzy Kukuczka Academy of Physical Education, Mikołowska 72a, 40 065 Katowice, Poland; 2 Department of Psychology, SWPS University of Social Sciences and Humanities, Chodakowska 19/31, 03-815 Warsaw, Poland; 3 Center for Kinanthropology Research, Faculty of Physical Culture, Palacký University Olomouc, Tř. Míru 115, 771 11 Olomouc, Czech Republic; 4 Department of Natural Sciences in Kinanthropology, Faculty of Physical Culture, Palacký University Olomouc, Tř. Míru 115, 771 11 Olomouc, Czech Republic

**Keywords:** Metabolic syndrome, Physical activity, Visceral fat, Women

## Abstract

This study was aimed at the evaluation of relationship between visceral fat area (VFA) and physical activity (PA) with the metabolic syndrome (MetS) risk in the physically active postmenopausal women. A total of 85 attendants of the University of the Third Age (U3A) aged 62.8 ± 5.9 years (median time since menopause 11.8 y), participated in this study. VFA was assessed by bioimpedance method using InBody 720 analyzer. PA was assessed using the ActiGraph GT1 M accelerometer. Fasting levels of serum lipids (TG, HDL), serum glucose, waist circumference (WC) and blood pressure were measured to diagnose MetS according to NCEP-ATP III criteria. In 73 out of 85 participants the VFA exceeded the upper normal level of 100 cm^2^, however, in almost a half of this group (*n* = 36) with elevated VFA (139.5 ± 26.1 cm^2^ on average), only 2 out of 5 criteria for MetS diagnosis were met. Participants were physically active, making on average 10,919 ± 3435 steps/day. The risk of MetS occurrence in women with VFA > 100 cm^2^ was twelve times higher (OR 12.33; CI 95% [1.5; 99.8]) than in the group with VFA < 100 cm^2^. The participants from the group with the highest PA level (≥12,500 steps/day) were at almost 4 times lower risk for MetS, than their less active counterparts (OR 3.84; CI 95% [1.27;11.64]). Increased level of VFA is a strong risk factor for the MetS in postmenopausal women, however high level of regular PA above the threshold of 12,500 steps/day may substantially reduce it.

## Introduction

There is a large scientific consensus that human aging is associated with changes in body composition, in particular, with accelerated loss of lean body mass and gains in body weight and body fat. Fat, a constituent of body mass, is a biologically active tissue contributing to cardiometabolic complications. The clinical and prognostic differences between subcutaneous and visceral fat tissues are significant, because visceral fat tissue is biologically more active than subcutaneous fat tissue (Fox et al. 2007; Kang et al. 2015). Age-related changes in adipose tissue distribution, indicating more truncal and less peripheral subcutaneous fat with older age (Després et al. 2008), may contribute to the development of the metabolic syndrome (MetS), a cluster of interrelated risk factors for cardiovascular disease (CVD) and diabetes, including obesity (particularly abdominal obesity), insulin resistance, hypertension, and dyslipidemia. One of the most widely used definitions of the metabolic syndrome is that created by NCEP ATP III (National Cholesterol Education Program (NCEP) Expert Panel on Detection, Evaluation, and Treatment of High Blood Cholesterol in Adults (Adult Treatment Panel III) 2002) which requires meeting at least three of five criteria, thus avoids any preconceived notion of the syndrome’s underlying cause, such as insulin resistance or obesity (Huang 2009). ATP III emphasized the importance of waist circumference (WC) as the estimate of adiposity on the premise that WC is more closely related to insulin resistance and its consequences than generalized obesity as indicated by body mass index (BMI) (Reaven 2005), however the most recent criteria accepted by the International Diabetes Federation (IDF) and the American Heart Association/National Heart, Lung, and Blood Institute (AHA/NHLBI) suggest that abdominal obesity is not a prerequisite of MetS, and the presence of any 3 of 5 risk factors are sufficient for its diagnosis (Alberti et al. 2009). Of note, there is much evidence that the incidence of MetS is related to individual hormonal status, and its prevalence is more markedly increased among women than in men, particularly over the age of 60 (Park et al. 2003; Rigo et al. 2009; Park and Kim 2015), as well as in postmenopausal compared with premenopausal women (Cho et al. 2008). Although the menopause itself is a period of normal biological change and adjustment, and not an illness, it promotes a change in body fat distribution, mainly an increase in central (or visceral) adiposity associated with enhanced probability of fulfilling the metabolic syndrome criteria (Bentley-Lewis et al. 2007). The factors contributing to the increased prevalence of MetS in postmenopausal women include also the declining level of estrogen, alterations in its ratio to testosterone, central adiposity and dyslipidemia (Janssen et al. 2008; Ziaei and Mohseni 2013; Peppa et al. 2013). Menopause is associated with changes in body fat distribution, especially with higher accumulation of visceral, than of subcutaneous fat. Visceral fat accumulation over the visceral fat area (VFA) cutoff point of 100 cm^2^ determined by computer tomography is considered responsible for the emergence of many adverse health problems (Piché et al. 2008).

Evidence-based guidelines for CVD prevention in women elaborated by AHA (Mosca et al. 2007; Bentley-Lewis et al. 2007) recommend several lifestyle interventions including cessation of smoking, diet control and exercise. If the visceral fat accumulation plays a key role in the development of metabolic syndrome, the lifestyle modification to reduce visceral adiposity would have priority over drug treatment (Matsuzawa et al. 2011).

The recommendations for adults include a minimum of 30 min of moderate intensity physical activity (PA) (brisk walking), preferably on most days of the week. Women determined to lose weight should accumulate a minimum of 60–90 min of moderate physical activity on most, and preferably on all days of the week (Mosca 2007). There is a general consensus that increasing physical activity helps to reduce body weight and body fat, so it has a beneficial effect on the metabolic risk factors, through lowering the overall ASCVD risk not only in general public (Franklin et al. 2004; Grundy et al. 2005; Garber et al. 2011), but also in older adults (Manini and Pahor 2009; Chodzko-Zajko et al. 2009), and specifically in older women (Mosca 2007; Mosca et al. 2007).

The present study is designed to determine the relationship between VFA assessed using bioimpedance analysis (BIA) and the metabolic syndrome risk in the physically active, postmenopausal women, all being active students of the local University of the Third Age (U3A) involved both in different activities offered by the U3A’s and in individually selected leisure time physical activities. This paper is an extension of our previous work (Zając-Gawlak et al. 2016), in which we presented a comparison of metabolic profiles of physically active U3A students with those recorded in a local, community dwelling cohort of individuals of the same age, selected from a nationwide PolSenior project. The aim of the current approach, including only the physically active female U3A students, was to evaluate the association of the VFA assessed by the bioimpedance analysis (BIA) with the metabolic syndrome risk in physically active postmenopausal women and to reveal whether the volume of physical activity may limit the risk of metabolic syndrome in women with elevated VFA content.

## Materials and methods

### Participants

A total of 85 postmenopausal (median time since menopause 11.8 years; median age at menopause onset 51.0 years) women aged 47–81 years (mean age 62.8±5.9 years), physically active female students of the local U3A Universities, volunteered to participate in this study. In addition to their routine daily activities, they were all involved in the physical activity program provided by the U3A’s. All participants signed informed consent before participating in the study, the protocol of which had been approved by the Ethics Committee.

### Assessment of the components of the metabolic syndrome

The metabolic syndrome (MetS) was diagnosed according to the NCEP/ATP III revised guidelines (Genuth et al. 2003; Grundy et al. 2004) in women who met three or more of the following criteria: (1) abdominal obesity as increased waist circumference (WC) ≥88 cm; (2) serum triglyceride level (TG) ≥150 mg/dl or being currently on drug treatment for high triglycerides; (3) high-density lipoprotein cholesterol level (HDL-C) <50 mg/dL or currently on treatment for low HDL-C; (4) systolic blood pressure (SBP) ≥130 mm Hg and diastolic blood pressure (DBP) ≥85 mm Hg or treated for hypertension; (5) fasting glucose level ≥100 mg/dl or using antidiabetic medication.

The WC was measured in the standing position to the nearest 0.5 cm at the midpoint between the lowest rib and the iliac crest. Visceral fat area (VFA), defined as a cross sectional area of visceral fat in the abdomen at the umbilical level (L_4_–L_5_), was estimated using multifrequency bioimpedance analysis (MF-BIA) with the InBody 720 analyzer (Biospace Co., Ltd., Seoul, Korea), considered a more convenient substitute method for measuring the VFA than computed tomography (Ogawa et al. 2011; Park et al. 2016). The measurements were taken under laboratory conditions, in line with the manufacturer’s instructions.

The participants attended to the laboratory between 8:00 and 10:00 a.m. in a fasted state for arterial blood pressure measurements and blood sampling. Blood pressure was measured twice after 15 min rest, using a standard mercury sphygmomanometer; the mean of two consecutive readings 2 min apart was taken for further analysis. Concentrations of serum glucose, high-density cholesterol (HDL-C), and triglycerides (TG) were assessed using enzymatic assays and commercially available diagnostic kits (Randox UK, Cat. No. GL 2623, CH 200, CH 203, TR 1697, respectively). Blood serum was separated using routine procedures and either processed immediately or kept frozen at −80 °C until analysis.

### Physical activity level

The PA levels of the participants were determined using the ActiGraph GT1M accelerometer (Manufacturing Technology Inc., FL, USA) after body composition testing. The devices were placed in the small pockets of elastic belts and were securely positioned near the right iliac crest of the participants, who were instructed to wear them for at least 12 h a day (an inclusion criterion) over eight consecutive days, excluding the time spent on water activities. To account for the effects of subject reactivity, the first day was omitted from data analysis. This allowed an objective evaluation of participants’ PA levels based on complete, 7-day accelerometer records (≥10 h of wear time a day) (Esliger et al. 2005). Having removed the accelerometers in the evening, the participants would record the times and types of PA and inactivity (i.e. sitting while watching TV, at the computer, at school, commuting) during the day. Physical inactivity is understood as unrecorded change of the ‘‘center of gravity’’ of the body, with up to 100 counts/minute, i.e. as a ‘‘stable body position’’ in a sitting, lying, or a different body position (Frömel et al. 2016). Data processing (30-s interval records) was performed using the Czech version of the software program IntPA13 (http://www.cfkr.eu). The time sampling interval of the accelerometers was set at 1 min, an epoch usually selected by users measuring free-living PA or epidemiological researchers (Esliger et al. 2005), and step mode was activated. Downloaded counts data were assessed and cleaned according to procedures proposed by Esliger et al. (2005). Because in the participants’ activity logs, swimming was classified as an activity when the accelerometer was not worn, this activity was subsequently assigned counts per minute. Participants were instructed to lead a normal lifestyle and to abstain from additional exercises. The measure of PA was the number of steps walked per day, with 10,000–12,449 steps/day being accepted as a universal step goal for classifying individuals as “active” and ≥12,500 steps/day as “highly active” (Tudor-Locke and Bassett 2004; Choi et al. 2007; Tudor-Locke et al. 2008; Aoyagi and Shephard 2010).

### Data analysis methods

Statistical analyses were performed using STATISTICA 12.5 software (StatSoft, Tulsa, OK, USA). For the ordinal-scale variables (PA levels, VFA levels) or continuous variables (fasting glucose, HDL-C, TG, SBP, DBP, WC, PA, height, weight, VFA, BMI) the basic statistical parameters were calculated (mean, median, standard deviations, extreme values) and then the variables were tested for normal distribution (the Shapiro–Wilk test). For the ordinal-scale variables and qualitative variables frequency distributions and percentage distribution were determined for particular categories of VFA, PA and MetS. To compare the number of MetS components met by participants with levels of visceral fat area (VFA: <100 cm^2^; 100–150 cm^2^; ≥150 cm^2^) and/or physical activity (PA: <10,000; 10,000–12,500; ≥12,500 steps/day), the Kruskal–Wallis analysis of variance and the pertinent post hoc tests were used. The odds ratios (and their 95% confidence intervals) for exceeded thresholds of VFA (≥100 cm^2^) or the number of steps (PA < 12,500 steps/day) as predictors of the risk of MetS were calculated.

## Results

**Fig. 1:**
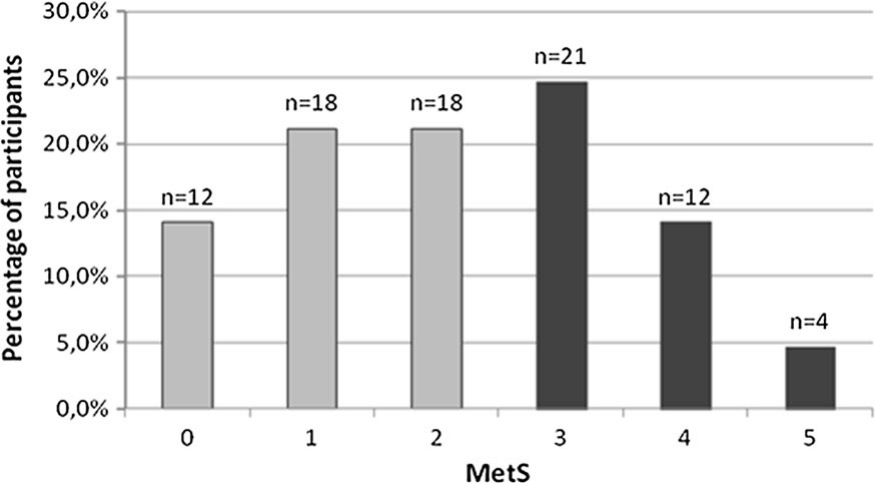
Percentages of participants (*N* = 85) meeting 0, 1, 2, 3, 4 or 5 criteria of the MetS. *Note* Median age of women stratified according to the number of MetS criteria met: 0–59.0 years; 1–60.2 years; 2–64.5 years; 3–62.0 years; 4–66.5 years and 5–61.8 years. Age-related trend *p* < 0.001

The descriptive statistics for all participants, further categorized into those without MetS (MetS criteria <3) or with MetS (MetS ≥3), are presented in Table 1. A preliminary analysis performed using the Mann–Whitney *U*-test showed that body height and age of menopause onset were the only variables that did not differentiate the sub-groups. Among all participants, only twelve women (15%) have not met any of the MetS criteria, while the remaining 73 women (85%) fulfilled at least one, and 37 of them (44%) were diagnosed as having the metabolic syndrome according to the NCEP/ATPIII criteria (Fig. 1). Of note, age and duration of menopause differentiated the sub-groups. Women with MetS were older and on average with longer time since menopause compared to their counterparts without MetS. Comparable results were obtained in previous studies (Park et al. 2003; Janssen et al. 2008; Park and Kim 2015).

**Table 1:** Baseline characteristics of women with and without the metabolic syndrome (mean ± standard deviation)—the Mann–Whitney *U* test

Variable	All women (*N* = 85)	No metabolic syndrome (*n* = 48) (MetS < 3)	Metabolic syndrome (*n* = 37) (MetS ≥ 3)	*p*
Age (years)	62.8 ± 5.9	61.3 ± 5.6	64.7 ± 5.9	0.008
Age at menopause onset (years)^a^	51.0 [49–54]	51.1 [50–54]	50.8 [48–58]	0.729
Duration of menopause (years)^a^	11.8 [7–16]	10.4 [5–14]	13.6 [8–18]	0.029
VFA (cm^2^)	137.7 ± 32.6	126.2 ± 32.7	152.7 ± 26.2	0.000
BMI (kg/m^2^)	27.6 ± 4.5	26.3 ± 4.5	29.2 ± 4.0	0.003
Height (m)	158.5 ± 5.2	158.9 ± 5.0	157.9 ± 5.4	0.396
Weight (kg)	69.2 ± 11.6	66.4 ± 11.7	72.8 ± 10.6	0.012
WC (cm)	85.3 ± 10.6	80.7 ± 10.2	91.4 ± 7.8	0.000
Fasting glucose (mg/dL)	92.2 ± 20.4	86.6 ± 11.6	99.4 ± 26.4	0.004
HDL-C (mg/dL)	62.7 ± 16.6	68.8 ± 14.4	54.8 ± 16.1	0.000
TG (mg/dL)	128.0 ± 46.8	106.7 ± 28.8	155.6 ± 51.3	0.000
SBP (mmHg)	132.2 ± 17.3	124.7 ± 15.9	142.0 ± 13.9	0.000
DBP (mmHg)	78.8 ± 10.6	76.3 ± 10.4	81.9 ± 10.2	0.016
PA (steps/day)	10,918.8 ± 3435.1	11,638.7 ± 3,797.3	9,985.0 ± 2,668.4	0.027
No of MetS criteria	2.2 ± 1.4	1.1 ± 0.8	3.5 ± 0.7	0.000

*VFA* visceral fat area, *BMI* body mass index, *WC* waist circumference, *HDL-C* high-density lipoprotein cholesterol, *TG* triglycerides, *SBP* systolic blood pressure, *DBP* diastolic blood pressure, *PA* physical activity (number of steps/day), *MetS* metabolic syndrome

^a^Since data about age at menopause onset and duration of menopause were not normally distributed, they are presented as median values plus their interquartile range (25–75th percentile)

### The risk of the metabolic syndrome (MetS) and the level of visceral fat area (VFA)

**Fig. 2:**
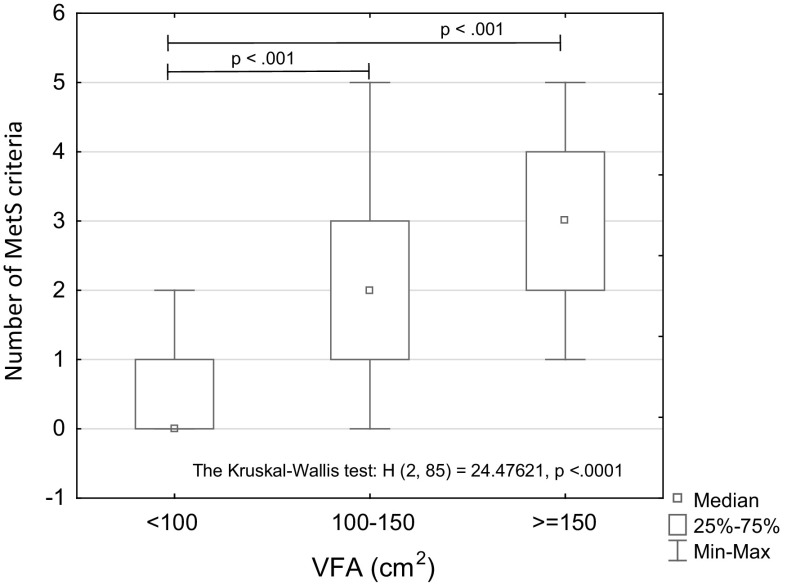
VFA levels and the number of MetS criteria met by the study participants. *Note* The number and median age of women stratified according to their VFA level: VFA < 100 cm^2^ *n* = 12; 58.5 years; VFA = 100–150 cm^2^-*n* = 43, 62.0 years; VFA ≥ 150 cm^2^-*n* = 30, 66.0 years. Age-related trend: *p* < 0.001

The mean value of the VFA calculated for all 85 participants exceeded the normal level (≥100 cm^2^). In 12 participants the VFA was within the normal (<100 cm^2^) and in 43 women—within the over normal range (100–150 cm^2^), while in the remaining 30 participants, the VFA level exceeded the extremely over normal limit (≥150 cm^2^) (Biospace 2004; Kim et al. 2011). The Kruskal–Wallis test revealed significant between-group differences in the number of MetS criteria diagnosed in study participants (H [2, 85] = 24.476, *p* < 0.001) (see Table 2; Fig. 2). Specifically, the post hoc tests revealed statistically significant differences in VFA between women with normal VFA and the groups with over normal and extremely over normal levels (p = 0.001 and **<**0.001, respectively). Moreover, there was a significant age-related trend (*p* < 0.001) toward higher levels of the VFA. Taking into consideration the prevalence of excess visceral adiposity VFA (in *n* = 73 out of 85 study participants) it is worth to note that MetS was diagnosed only in 19 out of 43 women with VFA 100–150 cm^2^ (44%) and in 15 out of 30 women with VFA >150 cm^2^ (50%) of the respective study groups.

**Table 2:** The number and the percentage prevalence of the metabolic syndrome (MetS) criteria in women with normal (N), over normal (ON) and extremely over (ExO) visceral fat area (VFA)

Group	VFA, cm^2^	The number of MetS criteria						Post hoc comparison between groups
		MetS < 3		MetS ≥ 3		The number of participants in relation to VFA level		
		*n*(%)	Mean ± SD	*n*(%)	Mean ± SD	*n*(%)	Mean ± SD	
Normal (N)	<100	12 (25.0)	0.5 ± 0.7	–	–	12 (14.1)	0.5 ± 0.7	N versus ON (*p* < .001) N versus ExO (*p* < .001) ON versus ExO (*p* = .099)
Over normal (ON)	100–150	24 (50.0)	1.2 ± 0.8	19 (51.4)	3.4 ± 0.6	43 (50.6)	2.1 ± 1.3	
Extremely over (ExO)	≥150	12 (25.0)	1.7 ± 0.5	18 (48.6)	3.7 ± 0,7	30 (35.3)	2.9 ± 1.2	

Kruskal–Wallis ANOVA: (H (2, 85) = 24.476, *p* < 0.001)

In order to assess whether there is an association between the level of the VFA and the risk of MetS, the odds ratio (OR) was calculated for study participants divided into two groups based on the cut-off value of the VFA (≥100 cm^2^), associated with an increase in the prevalence of the obesity-related disorders. The ratio showed that the group with over normal VFA was at 12 times higher risk for the metabolic syndrome (OR 12.33; CI 95% [1.5; 99.8]) compared to the group with normal VFA (Table 3).

**Table 3:** Odds ratios (95% Cl) for the metabolic syndrome (MetS) according to visceral fat area (VFA) and physical activity (PA) level

Number of MetS criteria	VFA ≥ 100 cm^2^				VFA < 100 cm^2^				OR	95% Cl
	*Mean*	*SD*	*n*	%	*Mean*	*SD*	*n*	%		
MetS < 3	1.33	0.72	36	49.32	0.50	0.67	12	100	12.33^+^	[1.5; 9.8]^+^
MetS ≥ 3	3.54	0.69	37	50.68	–	–	0 (→1)*	0		

Number of MetS criteria	PA < 12,500				PA ≥ 12,500				OR	95% Cl
	*Mean*	*SD*	*n*	%	*Mean*	*SD*	*n*	%		
MetS < 3	1.40	0.72	30	48.39	0.67	0.69	18	78.26	3.83	[1.21;11.64]
MetS ≥ 3	3.5	0.67	32	51.61	3.80	0.84	5	21.74		

*MetS* metabolic syndrome, *VFA* visceral fat area, *PA* physical activity (number of steps/day), *SD* standard deviation, *Cl* confidence interval, (−) criteria not met by any participant, *OR* odds ratio, (→1)* when the number of observations was 0, 1 was used to enable the calculation of the OR, but this reduced its value; (^+^) OR values calculated for 0 →1

### The risk of the metabolic syndrome (MetS) and the level of PA

**Fig. 3:**
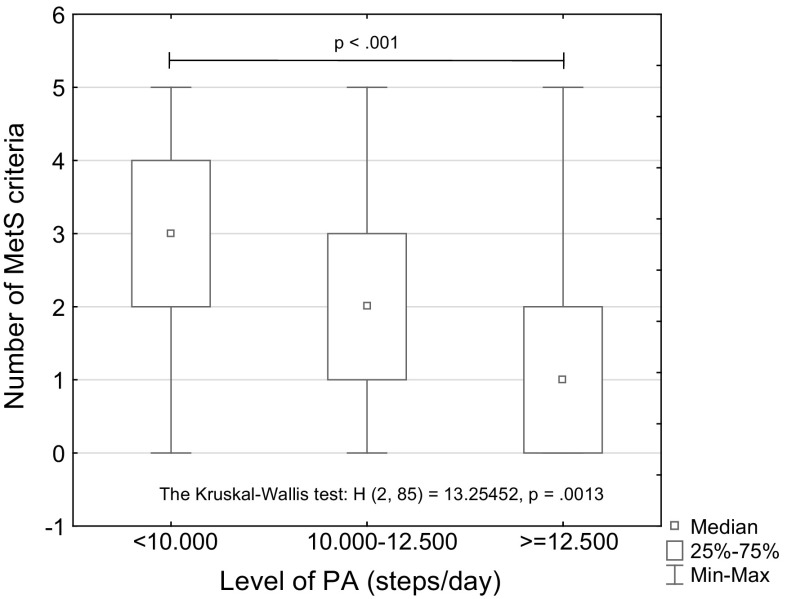
Physical activity levels (PA) and the number of MetS criteria met by the study participants. *Note* The number and median age of women stratified according to their PA level: PA < 10,000 steps/d-*n* = 38, 63.0 years; PA = 10,000–12,000 steps/days-*n* = 24, 63.0 years; PA ≥ 12,500 steps/d-*n* = 32, 59.0 years. Age-related trend: *p* < 0.05

The relationship between the objectively assessed physical activity (PA) level and the risk of the metabolic syndrome in our physically active attendants of the U3U’s was also investigated. The objectively assessed PA level, using Actigraph GT1M accelerometer, in the study participants’ revealed that 38 women took <10,000 steps per day, 24 participants—10,000 to 12,500 steps, and 23 most active women—took ≥12,500 steps per day. Therefore, they could be classified, respectively, as “somewhat active”, “active” and “highly active” (Tudor-Locke et al. 2008). The Kruskal–Wallis analysis of variance pointed to a statistically significant association between the number of MetS criteria and the daily number of steps (H [2.85] = 13.255, *p* = 0.0013; Table 4). According to the results of the post hoc tests, the number of MetS criteria in the most active women (≥12,500 steps/day) differed significantly (*p* = 0.001) from those diagnosed in their less active (<10,000 and 10,000–12,500 steps/day) counterparts. Notably, no statistically significant differences were found between the number of MetS criteria met by the least active (<10,000 steps/day) and active (10.000–12.500 steps/day) women (*p* = 0.552 and *p* = 0.131, respectively) (see Table 4; Fig. 3). Of note, significant differences in women age was noted between those who took <10,000 and ≥12,500 steps per day. Physical activity level appeared to have a significant impact on reduction of some components of the MetS, such as blood glucose, TG and HDL-C, that exceeded the normal levels, respectively, only in 19, 24 and 34 participants, i.e. in 22, 28 and 40% of the whole cohort. The remaining two MetS components (BP and WC) were less affected, as over normal levels were recorded, respectively, in 60 and 48 women (70 and 56% of the whole cohort). Of note, the mean PA leveI (10,918±3435 steps/day, see Table 1) was within the range of 8000–12,000 steps/day required for improvement in body composition parameters and specific health outcomes in women (Tudor-Locke et al. 2011a, b). However, only the participants from the group with the highest PA level (≥12,500 steps/day) were found to be at an almost four times lower risk for MetS than their less active counterparts (Table 3).

**Table 4:** The number and the percentage prevalence of the metabolic syndrome (MetS) criteria in women in relation to their physical activity (PA) level

Group	PA, steps/day	The number of MetS criteria						Post hoc comparison between groups
		MetS < 3		MetS ≥ 3		The number of participants in relation to PA level		
		*n*(%)	Mean ± SD	*n*(%)	Mean ± SD	*n*(%)	Mean ± SD	
1	<10,000	17 (35.4)	1.5 ± 0.6	21 (56.8)	3.6 ± 0.7	38 (44.7)	2.7 ± 1.2	1–3 (*p* < .001) 1–2 (*p* = .552) 2–3 (*p* = .131)
2	10,000–12,500	13 (27.1)	1.2 ± 0.8	11 (29.7)	3.3 ± 0.6	24 (28.2)	2.2 ± 1.3	
3	≥12,500	18 (37.5)	0.7 ± 0.7	5 (13.5)	3,8 ± 0.8	23 (27.1)	1.3 ± 1.5	

Kruskal–Wallis ANOVA: (H (2, 85) = 13.254, *p* = 0.0013)

In order to assess whether there is an association between the level of PA and the risk of MetS development, the odds ratio (OR) was calculated for participants divided into two sub-groups of the most active (PA ≥ 12,500 steps/day) and less active (PA < 12,500 steps/day) women. The ratio showed that women from the group with the highest PA level were at almost 4 times lower risk for the metabolic syndrome (OR 3.84; CI 95% [1.27;11.64]; Table 3) than their less active counterparts.

## Discussion

The results presented in this study, add some additional insights concerning the association of physical activity with VFA and risk of metabolic syndrome, into our earlier observations (Zając-Gawlak et al. 2016) on the effects of physical activity on body composition and metabolic profile in physically active postmenopausal women. There is substantial evidence supporting the notion that the excess of visceral adipose tissue, but not the amount of subcutaneous abdominal fat, is one of the most important anthropometric parameters to predict metabolic abnormalities associated with metabolic syndrome (Carr et al. 2004; Després et al. 2008; Matsuzawa et al. 2011). Some recent studies have evidenced that the VFA level over the cut-off value, and not WC cutoff value, is associated with the prevalence of MetS and is the single best predictor of MetS among women (Kim et al. 2011; Pickhardt et al. 2012). It is worth to note that high visceral fat area (VFA > 100 cm^2^) measured by the bioimpedance method using InBody 720 analyzer, was found in 73 women (86% of the cohort) (see Fig. 1). Such a high prevalence of study participants with increased central adiposity evidenced by over normal VFA level is most likely associated not only with their age, but also with their post-menopausal state, as the menopause does promote a change in body fat distribution to increase central adiposity (Bentley-Lewis et al. 2007; Cho et al. 2008; Janssen et al. 2008). In this context, important is our finding that women with over normal VFA level are at 12 times higher risk for metabolic syndrome compared with those without excess visceral adiposity (VFA < 100 cm^2^) (see Table 3). However, in our cohort of physically active postmenopausal women, the metabolic syndrome, according to the modified ATPIII criteria, was diagnosed only in 37 out of 85 participants (44% of the whole cohort) (see Table 2). This may imply that the over normal level of VFA does not require the presence of metabolic abnormalities typical for MetS, so the other differentiating factor may be involved.

It is well established that the risk of MetS can be modified by several lifestyle interventions, the most effective are increased physical activity and optimal dietary intake (Tudor-Locke et al. 2008; Aoyagi and Shephard 2010; Hayes et al. 2010). The most important finding of this study is that our highly active postmenopausal women taking more than 12,500 steps/day were found to be at almost 4 times lower risk of MetS (see Table 3) than their less active counterparts. At this point, it is well to recall the previously reported association of VFA level with the amount of PA expressed in steps/day (Spearman’s *R* = −0.507, *p* < 0.005) in this cohort of female U3A students (Zając-Gawlak et al. 2016). This can clearly confirm the beneficial effect of such a high level of physical activity on metabolic health even in older women with abdominal obesity. The literature data show that the threshold volume of habitual physical activity associated with health benefits should exceed the level of 7000–8000 steps/day. However, the threshold volume of habitual physical activity to prevent the development of MetS should be even greater, i.e. higher than 10,000 or 8000 steps/day for individuals aged 65–74 and 75–94 years, respectively. Considering that middle-aged and older women would prefer to choose walking for leisure time activity, the 10,000 steps/day recommendation is considered suitable for promoting health behavior changes in this population (Thompson et al. 2003; Krumm et al. 2006). Of note, additional health benefits may be further gained among individuals reaching daily step goal PA level >10,000 steps/day (Aoyagi and Shephard 2010), although important improvement in body composition parameters and specific health outcomes in women would require 8000 to 12,000 steps/day (Tudor-Locke et al. 2011a, b) or even more than 12,500 daily steps, the target achievable in postmenopausal women (Kroemeke et al. 2014).

In summary, the results of our study confirm generally accepted notion that increased level of VFA is a strong risk factor for the development of the MetS, and that a high level of physical activity above the threshold of 12,500 steps/day, achievable in a population of older, but highly motivated to be physically active, community dwelling women with over normal VFA, is associated with marked reduction in the MetS-related disorders.
